# Systematic Review of Solubility, Thickening Properties and Mechanisms of Thickener for Supercritical Carbon Dioxide

**DOI:** 10.3390/nano14120996

**Published:** 2024-06-07

**Authors:** Xiaohui Wang, Qihong Zhang, Shiwei Liang, Songqing Zhao

**Affiliations:** 1 Beijing Key Laboratory of Optical Detection Technology for Oil and Gas, China University of Petroleum (Beijing), Beijing 102249, China; wangxiaohui@cup.edu.cn (X.W.); 2021211324@student.cup.edu.cn (Q.Z.); 2023211325@student.cup.edu.cn (S.L.); 2 National Key Laboratory of Petroleum Resources and Engineering, China University of Petroleum (Beijing), Beijing 102249, China; 3 State Key Laboratory of Heavy Oil Processing, China University of Petroleum (Beijing), Beijing 102249, China

**Keywords:** supercritical CO_2_, thickener, intermolecular interaction, functionalized polymer

## Abstract

Supercritical carbon dioxide (CO_2_) has extremely important applications in the extraction of unconventional oil and gas, especially in fracturing and enhanced oil recovery (EOR) technologies. It can not only relieve water resource wastage and environmental pollution caused by traditional mining methods, but also effectively store CO_2_ and mitigate the greenhouse effect. However, the low viscosity nature of supercritical CO_2_ gives rise to challenges such as viscosity fingering, limited sand–carrying capacity, high filtration loss, low oil and gas recovery efficiency, and potential rock adsorption. To overcome these challenges, low–rock–adsorption thickeners are required to enhance the viscosity of supercritical CO_2_. Through research into the literature, this article reviews the solubility and thickening characteristics of four types of polymer thickeners, namely surfactants, hydrocarbons, fluorinated polymers, and silicone polymers in supercritical CO_2_. The thickening mechanisms of polymer thickeners were also analyzed, including intermolecular interactions, LA–LB interactions, hydrogen bonding, and functionalized polymers, and so on.

## 1. Introduction

In recent years, carbon dioxide (CO_2_) has become the focus of carbon emission reduction. As a major greenhouse gas, the utilization and storage of CO_2_ (as a buffer gas when producing hydrogen) are required to be environmentally friendly and economically sustainable [1,2,3,4,5,6]. For the chemical properties of CO_2_, the carbon atoms in the CO_2_ molecule are hybridized in *sp* mode, and the electrons form two mutually perpendicular π bonds. The bond length of the carbon–oxygen double bond (O=C=O) is shorter than that of the carbonyl bond (C=O). Thus, the CO_2_ structure is stable and the chemical properties are not very reactive [7], and the phase diagram of CO_2_ can be seen in Figure 3 of Ref. [8]. The critical points of CO_2_ are at 31 °C and 74 bar [8]. When the temperature and pressure exceed the critical point, CO_2_ undergoes a transition into the supercritical state, resulting in the formation of supercritical CO_2_.

Supercritical CO_2_ is a substance that exhibits properties intermediate between those of a gas and a liquid. It possesses advantageous characteristics such as high diffusivity, low viscosity, low surface tension, and controllable solubility. This unique nature of supercritical CO_2_ finds extensive applications in oil displacement technology and fracturing technology [9], effectively addressing the limitations associated with hydraulic fracturing [10,11]. These limitations include excessive water consumption, clay swelling, reservoir damage caused by residual working fluids, and inadequate flowback leading to groundwater pollution [12,13]. However, the low viscosity characteristics of pure supercritical CO_2_, with a viscosity of only 0.02–0.05 mPa·s, will cause a series of problems including viscous fingering, limited sand–loading capacity, and filter damage. The viscosity fingering problem stems from the viscosity difference between supercritical CO_2_ and crude oil, which causes supercritical CO_2_ to form finger–like flows in the reservoir, bypassing the oil layer and reducing recovery efficiency [14]. High filtration loss means that part of the fracturing fluid is adsorbed, retained, and permeated into the formation during the fracturing process. The problem of limited sand–carrying capacity is due to the low viscosity and high diffusivity of supercritical CO_2_, which limits its ability to carry sand particles and affects the effective support of fractures [10,15,16,17].

Therefore, understanding how to increase the viscosity of supercritical CO_2_ has become extremely important. The most direct and effective method is to add a thickener to supercritical CO_2_. The ideal supercritical CO_2_ thickeners should be effective in increasing viscosity at low doses. From a quantitative point of view, the thickening ratio is sufficient when it can thicken supercritical CO_2_ 200–300 times, which is achieved in fluorinated thickeners [18], yet fluorinated thickeners are toxic, limiting their on–site application. In principle, the higher the viscosity of supercritical CO_2_, the better it would fit within the range to be achieved. In oilfield applications, the ideal efficiency of thickening depends on the actual application needs. For a broad range of applications, the increase of 5 to 10 times will serve the purpose. For oil extraction in the Middle East, a viscosity increase of 10 times is more than adequate. And in the context of supercritical CO_2_ fracturing technology, the viscosity enhancement should range from 20 to 30 times the original value. In addition to an increase in viscosity, an ideal thickener should also be cheap, environmentally friendly, safe, high efficiency, and soluble in supercritical CO_2_ but insoluble in water [19]. At present, supercritical CO_2_ thickeners are generally divided into the following four categories: surfactants, hydrocarbon polymers, fluorinated polymers, and silicone polymers [20]. In particular, fluorine–containing thickeners have the best solubility and thickening effects, but their use is restricted as they are expensive and not environmentally friendly [21,22]. Furthermore, they are not effective at low concentrations and also adsorb to rock [23]. This article mainly reviews the research progress of the four types of thickeners in terms of solubility, thickening properties, and mechanisms.

## 2. Characterization Parameters of Solubility and Thickening Properties

Through much literature research, it can be seen that the ideal supercritical CO_2_ thickener requires both efficient solubility and thickening properties in CO_2_ without any additional cosolvents. The characterization parameter of solubility and thickening properties are summarized below.

### 2.1. Solubility Properties

In terms of solubility, the main influencing factors are the interaction between the polymer and CO_2_ molecules, solvent CO_2_ density, solute relative molecular mass and molecular polarity, and especially the density factor. Solubility increases exponentially with the density of a supercritical CO_2_ system [24]. For thickening properties, it is mainly influenced by the spatial network structure formed by the interaction between thickener molecules. This structure effectively impedes the flow of CO_2_ molecules, resulting in the thickening of supercritical CO_2_.

The dissolving properties of thickeners in supercritical CO_2_ can be described by the solubility parameter δ [25], which is equal to the arithmetic square root of the cohesive energy density. The closer the polymer solubility parameter is to the CO_2_ solubility parameter, the better the solubility in CO_2_. The addition of cosolvents can reduce differences in the solubility parameters [25].

According to Enick’s research on thermodynamics, in order to be dissolved in supercritical CO_2_, Gibbs free energy (*G_mix_*) must be reduced, that is Δ*G_mix_* < 0 [26], and its related expression is as follows:

(1) $$∆G_{mix}=∆H_{mix}-T∆S_{mix}$$

Here, Δ*H_mix_*, Δ*S_mix_*, and *T* are the mixing enthalpy change, mixing entropy change, and absolute temperature, respectively. Therefore, the above problem is transformed into the problem of how to improve Δ*H_mix_* and Δ*S_mix_*.

For *H_mix_*, the main influencing factors are the density of the CO_2_ mixed solution, the interaction between CO_2_ molecules, between the polymer thickener molecules, and between the polymer thickener and CO_2_ molecules. However, the interaction between the polymer thickener and the CO_2_ molecules is critical in determining whether the thickener can be dissolved in liquid CO_2_. This interaction effectively promotes enthalpy reduction, which in turn reduces the free energy, allowing the thickener to dissolve in supercritical CO_2_ [27]. The strength of the interaction between molecules can be described by cohesive energy density [25]. For CO_2_ molecules, the electron distribution is near oxygen atoms and the CO_2_ molecule has a zero dipole moment; however, a large quadrupole moment and low polarizability [28] can cause weak interactions with non–polar covalently bonded fragments like C–C motifs, but reasonably strong interactions with non–hydrogen–bonded polar functional groups like esters, ethers, C–F groups, or aromatic structures. Therefore, in order to be dissolved in CO_2_, it should be weakly polar and have low cohesive energy density or contain a certain number of CO_2_–philic groups.

As for Δ*S_mix_*, the solubility of the polymer can be improved by increasing entropy of the system. For example, increasing the free volume of the polymer, improving the flexibility of the polymer chain, and lowering the glass transition temperature can all help reduce the interaction between polymer molecules and thus promote dissolution. Additionally, increasing the degree of branching of the polymer thickener can have a similar effect [29].

The interaction between the polymer and CO_2_ is one of the key factors determining the solubility of the polymer. If the interaction energy between the polymer and CO_2_ is strong, it usually means that the solubility of the polymer in supercritical CO_2_ is better because this strong interaction helps to overcome the attraction between the polymer molecules or between the CO_2_ molecules such that the thickener can be dispersed into the solvent. The interaction energy between the polymer and the CO_2_ molecules provides a measure of the strength of this interaction and can be calculated via Formula (2) [30].

(2) $$E_{inter}=E_{polymer-{CO}_{2}}-\left(E_{polymer}+E_{{CO}_{2}}\right)$$

The total energy *E_CO_*_2_ of supercritical CO_2_, the total energy *E_polymer_*, and the total energy *E_polymer–CO_*_2_ of the mixed system can be calculated through molecular dynamics (MD). The larger the absolute value of *E_inter_*, the stronger the interaction. Hu et al. used MD methods to study poly(vinyl acetate–alt–maleate) copolymers. The results show that this type of polymer reduces the interaction energy between the polymer and CO_2_ after copolymerizing vinyl acetate, but it remains higher than the interaction energy between PVAc and CO_2_ [31].

In addition, the adsorption of polymers in supercritical CO_2_ can be reflected by the potential of mean force (PMF), which is calculated as shown in (3) [32]. The change in PMF can reflect the positional preference of polymers in CO_2_. If the PMF value is negative, it indicates that the polymer is more stable at a certain position in supercritical CO_2_, which contributes to polymer solubilization.

(3) $$E_{\left(r\right)}=-k_{B}T\ln g_{\left(r\right)}$$

where *E*_(*r*)_ is the mean force potential, *k_B_* is Boltzmann’s constant, *T* is the absolute temperature, *g*_(*r*)_ is the radial distribution function between the polymer and CO_2_. The physical significance of *g*_(*r*)_ can be expressed as the ratio of the local density of the B atom to the intrinsic density of the A atom at a distance *r* from the central atom A. This indicates that CO_2_ molecules need to overcome a certain barrier to approach the thickener. Moreover, the solubility of polymers in CO_2_ is also affected by polymer–polymer interactions to the same degree. The weaker the polymer–polymer interaction and the stronger the polymer–CO_2_ interaction, the more favorable the solubility.

Among the interaction between the polymer and CO_2_, Lewis acid–base (LA–LB) interaction can effectively promote dissolution of polymer [25]. In an LA–LB interaction, the Lewis acid acts as an electron pair acceptor and can accept electron pairs, while the Lewis base acts as an electron pair donor and can donate electron pairs. For example, Gong et al. [25] found that the LA–LB interaction between O atoms in PVAc and C atoms in CO_2_ can enhance the solubility of PVAc in supercritical CO_2_. This interaction helps more CO_2_ molecules distribute around the carbonyl groups in the PVAc molecular chain, thereby increasing the solubility of PVAc in supercritical CO_2_.

### 2.2. Thickening Properties

The viscosity of the mixed solution can be calculated by the following formula [33].

(4) $$\eta =\frac{\tau_{w}}{\gamma_{w}}=\frac{D\Delta p/4L}{8\nu /D}$$

where *η*(Pa·s) is the viscosity, *τ*_w_ and *γ*_w_ are the wall shear stress, and apparent shear rate, relatively. *D* is the capillary diameter, ∆*p* is the capillary pressure difference, *L* is the capillary length, and *ν* is the flow rate of CO_2_ in the thickened liquid. Research shows that the thickened supercritical CO_2_ system is a non–Newtonian fluid and the viscosity has a non–linear relationship with the shear rate [33]. Moreover, the viscosity can also be acquired by fitting the following transverse current autocorrelation function below:

(5) $$C_{⊥(k,t)}\sim e^{-\frac{\eta k^{2}}{\rho }t}$$

which can be obtained through MD simulation [32].

## 3. Supercritical CO_2_ Thickeners

### 3.1. Surfactants

Surfactant thickeners are polymers composed of nonpolar hydrophilic groups and polar hydrophobic groups [34,35]. In supercritical CO_2_, the hydrophilic groups of the surfactant undergo physical interactions with a small amount of water, while the hydrophobic groups are exposed to and interact with CO_2_, forming a reverse micelle structure that further develops into a spatial network structure [36,37]. This network structure can restrict the mobility of CO_2_ molecules, thereby serving a thickening function.

The solubility of the polymer ZCJ–01 (copolymers of styrene with modified sulfonated fluorinated acrylates), surfactant thickener APRF–2 (consists of sodium succinate (–2–ethyl) sulfonate, ethanol, and H_2_O, etc.) and surfactant thickener SC–T–18 in supercritical CO_2_ was investigated by Zhai et al. [38]. The main component of SC–T–18 is a comb copolymer with polydimethylsiloxane as the main chain and amino groups as side chains. The research results show that SC–T–18 has the highest solubility among them, and the experimental values are in good agreement with the theoretical values [38]. SC–T–18 and supercritical CO_2_ form a single, stable, homogeneous emulsion micelle after sufficient mixing, which is due to the side–chain amino group in SC–T–18 effectively increasing its solubility in CO_2_ through an LA–LB interaction. At 25 °C and 6.894 MPa, Enick [39] conducted a study on the use of a 1 wt% surfactant tributyltin fluoride thickener and a 40–45 wt% pentane cosolvent to increase the viscosity of the supercritical CO_2_ system. The results showed that this combination can thicken the viscosity by 10–100 times. However, the flaw of this thickener is the necessity to use a considerable number of cosolvents to facilitate its dissolution in supercritical CO_2_, which makes the process very inefficient. Considering that the addition of cosolvents will bring more serious environmental problems, Shi et al. [40,41,42] introduced a fluoroalkyl group into the trialkyltin fluoride polymer molecule to obtain a semi–fluorinated trialkyltin fluoride thickener. Research shows that this thickener has high solubility in supercritical CO_2_ systems without the use of any cosolvents. Under 10–18 MPa, 4% mass fraction of semi–fluorinated trialkyltin fluoride can increase the system viscosity by up to 3.3 times. Enick et al. [43] also used perfluoropolyether glycol and fluorinated diisocyanate to react to synthesize a fluorinated polyurethane thickener. At 25 °C and 25 MPa, 4% fluorinated polyurethane can increase the viscosity of the system by 1.7 times.

**Figure 1:**
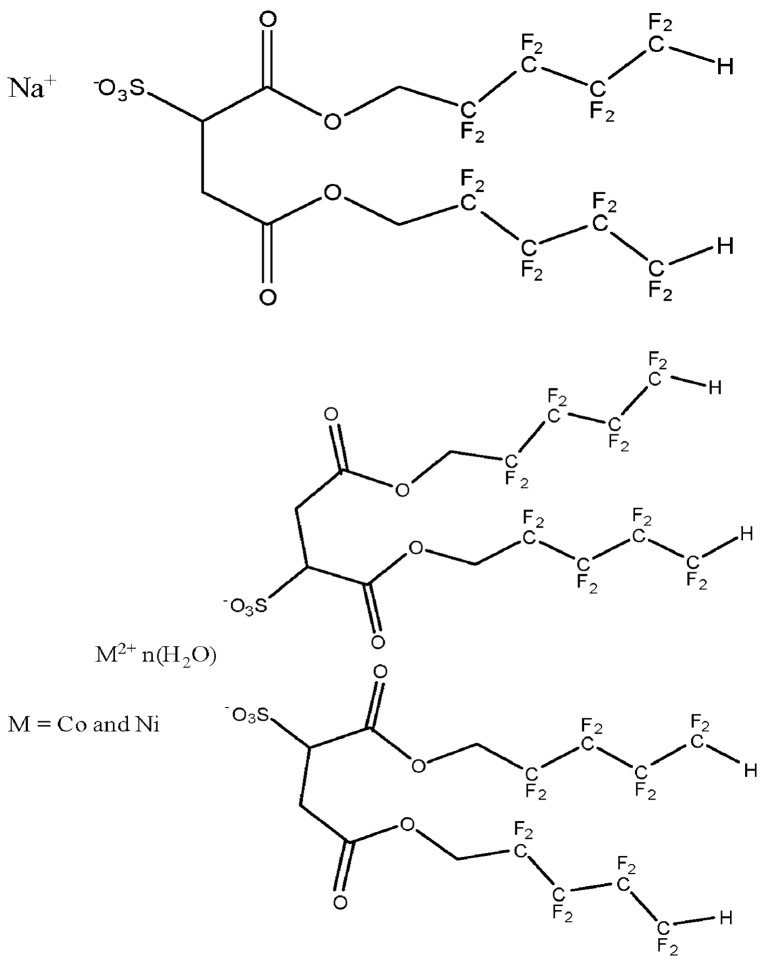
Structures of Na^+^(di–HCF_4_) and M^2+^(di–HCF_4_). Reprinted (adapted) with permission from [44]. Copyright© 2010, American Chemical Society.

Trickett et al. [44] designed a surfactant that can not only dissolve in CO_2_, but also form rod–shaped micelles with enhanced viscosity when a small amount of water is added. They changed Na^+^ in dialkyl sulfosuccinic acid into M^2+^ (Co^2+^ or Ni^2+^), as shown in Figure 1, turning the spherical micelles into rod–shaped micelles, and then forming reverse micelles in supercritical CO_2_. Then, through the interaction between these micelles, the viscosity of the CO_2_ mixed system increased. Research results show that Co(di–HCF_4_)_2_ and Ni(di–HCF_4_)_2_ with 6–10 wt% can increase the viscosity of the CO_2_ mixed system by 20–90 wt%. For some surfactant thickeners that are difficult to dissolve in supercritical CO_2_, CO_2_–philic groups can be introduced, such as fluorinated amine– and oxygen–containing surfactants [45]. Semi–fluorinated and fluorinated surfactant thickeners are found to be soluble in CO_2_ liquids and can also increase CO_2_ viscosity through the addition of a small amount of water [46]. By studying the solubility of oxygenated hydrocarbon surfactants in CO_2_, it was found that this thickener has a similar level to that of fluorinated surfactants, and both show high solubility properties, indicating that the O atoms in oxygenated hydrocarbon surfactants can increase solubility [47].

The principle of surfactant thickening of CO_2_ involves the formation of reverse micelles by surfactants. These reverse micelles overlap and entangle with each other, creating a spatial network structure. This structure restricts the flow of CO_2_ molecules, resulting in the thickening effect of the system. In addition, hydrocarbons, polar, or ionic groups can also be introduced into surfactants to increase the viscosity of supercritical CO_2_ systems [48]. The interaction between ion charges and water dipoles, as well as the Van der Waals force between alkyl chains, are also important factors in the formation of ionic surfactant micelles. The former is required to be stronger than the interaction between ions and CO_2_, and as for the latter, specific functional groups can be introduced to enhance the Van der Waals forces. In order to enhance the solubility of thickener molecules in supercritical CO_2_, CO_2_–philic groups, such as fluoroalkyl groups, carbonyl groups, and oxygen atoms, can be introduced into surfactant thickener molecules.

### 3.2. Hydrocarbon Polymers

Generally, this type of thickener contains carbon (C), hydrogen (H) elements, and may contain oxygen (O) elements. Hydrocarbon polymer thickeners usually have low solubility and weak thickening properties. Heller et al. [49] conducted a study on commercially available hydrocarbon polymer thickeners and found that the viscosity of the supercritical CO_2_ system did not significantly increase through the introduction of hydrocarbon polymer thickeners. They discovered that only a portion of the thickeners was soluble in supercritical CO_2_. The solubility of this portion was attributed to the polymers’ amorphous and irregular structure, which lacked a compact crystalline arrangement. This structural characteristic allowed for greater space, facilitating the penetration and solubilization of CO_2_ molecules. Not only that, the solubility of this type of polymer in supercritical CO_2_ was influenced by the interactions between the polymers, as well as between the polymers and CO_2_. The solubility is higher when the interaction between the polymer and CO_2_ is stronger.

Sarbu et al. [50] believed that polymers soluble in CO_2_ should have monomer units of LA–LB interaction with CO_2_ and monomer units with high free volume and high flexibility. Shen et al. [51] confirmed that among all the hydrocarbon thickeners, polyvinyl acetate (PVAc, Mw = 125,000) has the best solubility in supercritical CO_2_. The reason is that PVAc contains acetic acid groups, which can effectively increase solubility, but its ability to thicken CO_2_ is relatively weak. Because of this, PVAc has become an ideal supercritical CO_2_ thickener design material. Shen et al. [52] used azobisisobutyronitrile (AIBN) as a catalyst to synthesize a polyvinyl acetate telomer through free radical reaction, and then polymerized it with styrene to form a binary copolymer, namely styrene vinyl acetate binary copolymer, and the reaction mechanism is shown in Ref. [52] (1), (2) and (3). This copolymer molecule has CO_2_–philic groups and thickening groups, namely acetic acid groups and styrene groups, respectively, and is expected to become an economical and environmentally friendly thickener. Zhang Jian [53] used AIBN as an initiator to synthesize four–arm PVAc through the reversible addition–fragmentation–chain transfer (RAFT) polymerization method. In the case of adding cosolvent ethanol, at 35 °C and 15 MPa, adding four–arm PVAc with a concentration of 1 wt% and ethanol with a concentration of 5 wt% can increase the viscosity of the supercritical CO_2_ system by 31–55%. However, this still does not meet the standards for actual use.

In recent years, MD simulations have been used to study the solubility of supercritical CO_2_ and the thickening properties of thickeners. Xue et al. [32] used MD simulation methods to calculate the thickening mechanism of supercritical CO_2_ thickener and found that polyvinyl acetate–copolyvinyl ether (PVAEE) molecular chains formed a spatial network structure by intertwining with each other. Due to the interaction between CO_2_ molecules and polymers (including electrostatic interactions and Van der Waals interactions), the polymer restricted CO_2_ molecules to the network structure formed by PVAEE (degree of polymerization of each chain was N = 50) molecular chains. This restriction reduced the supercritical CO_2_ molecules flow, thereby increasing the viscosity of the supercritical CO_2_ fluid. They also obtained PMF by calculating the radial distribution function (RDF) in the results. The contact minimum (CM) and the second solvent separation minimum (SSM) are 0.4 nm and 0.85 nm, respectively. Their corresponding energy values determine the binding stability of CO_2_ and the polymer in the first and second solvent layers, respectively. A higher energy barrier exists between the CM and the SSM, which is the barrier of the solvent layer (BS). The equations for calculating the binding energy barrier and dissociation energy barrier between CO_2_ and polymer groups are ∆*E*^+^ = *E*_BS_ − *E*_SSM_ and ∆*E*^−^ = *E*_BS_ − *E*_CM_, respectively. The corresponding binding and dissociation energy barriers for the ether (ester) groups are 700 kJ/mol and 300 kJ/mol (540 kJ/mol and 190 kJ/mol), respectively. These values indicate that the ester group is more readily bonded to CO_2_. Goicochea et al. [54] also used MD simulation to study the interaction between polymers and CO_2_ molecules. Research shows that both intermolecular interactions and branching can improve the viscosity of supercritical CO_2_. In particular, intermolecular π–π stacking plays a crucial role in the thickening effect of supercritical CO_2_. These studies show that MD simulation is a very effective method to study supercritical CO_2_ systems at the molecular level.

Double–chain polyether carbonate (TPA–PEC, Mw = 2168, N = 30), tri–chain polyether carbonate (TMA–PEC, Mw = 2211, N = 30), and four–chain polyether carbonate (TFA–PEC, Mw = 2254, N = 30) were synthesized as CO_2_ thickeners by Chen et al. [55]. The respective dissolution properties were studied using the MD simulation method at 24.85 °C and 20 MPa. The results show that both TPA–PEC and TMA–PEC have better solubility than TFA–PEC due to their stronger interaction with the CO_2_ molecules, but their thickening effect is poor. TFA–PEC has the highest viscosity and only needs an addition of 0.95 wt% to thicken the supercritical CO_2_ viscosity by 11 times, while TMA–PEC needs to be added at 0.72 wt% to thicken the CO_2_ viscosity by 3.9 times. Among the three, TPA–PEC has the worst CO_2_ thickening ability. From the perspective of solubility and thickening properties, the multi–chain structure is beneficial to the thickening ability but not to solubility. Polyether carbonate is also a polymer thickener that is easily degraded under natural conditions and has the advantage of being environmentally friendly.

Afra et al. [23] investigated the supercritical CO_2_ system of Poly–1–decene (P1D, Mw = 2950) in sandstone rock properties. It was shown that 1.5 wt% P1D increased the viscosity of the supercritical CO_2_ system by a factor of six at 24.13 MPa and 35 °C. When the temperature was increased to 90 °C, the viscosity increased by a factor of 4.8. It was also shown that the large number of methyl groups in the P1D molecule contributes to its solubility in CO_2_, while the branched structure of the molecule positively affects the thickening effect as well. In addition, poly–1–decene (an oligomer of about 20 repeating units) is not only very effective in CO_2_ viscosification, but also reduces the remaining water saturation from 40 to about 27% at 24.13 MPa and 90 degrees celsius, which can improve the storage efficiency of CO_2_.

Sun et al. [56] used AIBN as the initiator and synthesized a series of copolymers P(HFDA–co–MMA) and P(HFDA–co–EAL) using HFDA (1H,1H,2H,2H–perfluorodecyl acrylate), EAL (ethyl acrylate), and MMA (methyl methacrylate), as shown in Ref. [56] Scheme 1. The microstructure and intermolecular interactions in the supercritical CO_2_ system were studied through MD simulations. According to their research, an increase in the content of the EAL group enhances the interaction between copolymer chains and reduces their flexibility, leading to a decrease in solubility. Moreover, the intermolecular association of the copolymer is strengthened, resulting in an increased thickening ability. At 35.05 °C and 30 MPa, the P(HFDA_0.19_–co–EAL_0.81_, Mw = 3576) copolymer with the highest EAL content increases the viscosity of supercritical CO_2_ by 96 times at 5 wt% concentration and has the best thickening property among all copolymers. The solubility and thickening properties of P(HFDA_0.37_–co–EAL_0.63_) are higher than those of P(HFDA_0.39_–co–MMA_0.61_). P(HFDA_0.37_–co–EAL_0.63_, Mw = 3394) increases the viscosity of supercritical CO_2_ by 70 times, while P(HFDA0.39–co–MMA0.61) only increases it by 40 times. It shows that although EAL and MMA are isomers, the differences in their structures and compositions make huge differences in the intermolecular interactions of copolymer–CO_2_ and the association between copolymer chains. The presence of methyl groups in the main chain of P(HFDA–co–MMA) increases steric hindrance, which reduces intermolecular association, free volume, and chain flexibility.

Furthermore, at 344.3 K and 25–45 MPa, a coarse–grained molecular modeling study optimized by Kazuya [18,57] via the particle swarm optimization algorithm showed that branched hydrocarbon poly–1–decene oligomers (especially the model with six repeating units and Mw = 1000) showed a significant increase in solubility in supercritical CO_2_ compared to straight–chained alkanes with the same molecular weight, up to a factor of 270 times. This increase is attributed to an increase in the number of branches in the molecular structure, especially structural edges (methyl groups), which have enhanced interactions with CO_2_ and thus increase solubility. The branched structure of the thickener not only increases its solubility in CO_2_ but also reduces the adsorption of the thickener to the rock as compared to the change in chemical composition [57]. These findings provide important molecular design principles for the development of thickeners with high solubility in supercritical CO_2_. Ding et al. [58] conducted a study on the solubility and thickening properties of oligomers of 1–decene (O1D) with six repeat units and oligomers with branches of 1–dodecene and 1–hexadecene (O1D1H). The research confirmed that branches and methyl groups can promote solubility. At approximately 13.8 MPa and 35 °C, the solubility of O1D in supercritical CO_2_ is 0.6 wt%. While at 24.1 MPa and 35 °C, the solubility of O1D1H is 0.3 wt%. However, in terms of relative viscosity, the 0.3 wt% concentration of O1D1H provides better viscosity performance than the 0.6 wt% concentration of O1D.

In general, the biggest problem with hydrocarbon polymer thickeners is their low solubility and difficulty in completely dissolving in supercritical CO_2_. At present, the main way to quickly solve the problem of solubility is to add a large amount of cosolvent, but this also has the implication of environmental problems caused by the cosolvent, which is neither economically nor environmentally friendly. Therefore, it is still necessary to modify and design the hydrocarbon thickener molecules themselves to find thickeners with high solubility and high thickening properties.

### 3.3. Fluorinated Polymers

Compared with hydrocarbon polymer thickeners, fluorinated polymer thickeners obtained after fluorination have stronger CO_2_–philic properties and can be effectively dissolved in liquid CO_2_ without adding cosolvents. At the same time, the polymer has a better thickening effect.

DeSimone et al. [59] demonstrated for the first time that fluoropolymers can be strongly dissolved in supercritical CO_2_ without the assistance of cosolvents and show good thickening properties. Research shows that at 50 °C and 300 bar, 3.7 wt% poly(1,1–dihydroperfluorooctyl acrylate) (PFOA, Mw = 1,400,000) in supercritical CO_2_ can increase the viscosity of the system from 0.08 cP to 0.20–0.25 cP. However, PFOA is toxic to aquatic organisms, which may cause disruption to aquatic ecosystems.

Huang et al. [60] synthesized copolymers (PolyFAST) using a fluorinated acrylate and styrene copolymer. Among them, the fluorocarbon group is CO_2_–philic and can improve the solubility of PolyFAST in supercritical CO_2_, while the styrene group is CO_2_–phobic and can thicken CO_2_ but can also reduce the solubility of PolyFAST in supercritical CO_2_. The ratio of fluorinated acrylate to styrene is crucial in determining the thickening effect. Through multiple experiments, it has been found that the most significant increase in viscosity occurs when using a ratio of 71 mol% fluorinated acrylate and 29 mol% styrene [60]. Additionally, incorporating 1–5 wt% PolyFAST in supercritical CO_2_ can result in viscosity increasing up to 5–400 times. However, the production cost of this polymer is relatively high, and it is not environmentally friendly.

Heller et al. [49] studied telechelic polymer thickeners which have corresponding ionic groups at each end and form a network structure in the form of ion pair aggregation. Enick et al. [61] synthesized poly–sulfonated polyurethane. Fluorinated telechelic ionic polymers have good solubility in CO_2_ and the addition of 4 wt% the polymer can increase the viscosity of CO_2_ by 2.7 times at 25 °C and 25 MPa.

Shi et al. [42] synthesized a series of semi–fluorinated trialkyl tin fluorides. Among them, 4 wt% tris(1,1,2,2–tetrahydroperfluorohexyl)tin fluoride is soluble in CO_2_ and can increase the thickening of the supercritical CO_2_ system by 3.3 times. The mechanism is that the positively charged Sn atoms and the negatively charged F atoms form Sn–F bridges to create transient polymer chains.

Sun et al. [62] used an all–atom MD to simulate the molecular model of the polymer–CO_2_ system and studied the solubility and thickening properties of the copolymer in supercritical CO_2_. Research shows that 5 wt% of P(HFDA_0.49_–co–VPc_0.51_, Mw = 3023) can increase the viscosity of the CO_2_ system by 62 times. The thickening performance of 1.5 wt% of P (HFDA_0.31_–co–VAc_0.69_, Mw = 3539) is higher than that of P (HFDA0.49–co–VAc0.51) under the same conditions, but it is not easily dissolved at higher concentrations. The main reason is the high concentration of VAc which increases the number of methyl groups in the polymer chain, resulting in a decrease in chain flexibility. Therefore, the length and composition of polymer side chains can greatly affect the thickening performance.

Huang zhou [27] synthesized a CO_2_ thickener containing a fluoro–urea group. Research shows that DCT ([1,6–Bis(1,3–diperfluorooctanoic acid propyl–2–ureido)]heptane, double–chain thickener) begins to thermally degrade at around 200 °C, and SCT ((1,6–Difluorooctanoic acid ethyl ester urethyl)hexane, single–chain thickener) begins to thermally degrade at 170 °C. The optimal mass fraction obtained through single–factor experiments is 2 wt%, and DCT makes the viscosity of supercritical carbon dioxide is increased to 1.54 mPa·s, while the SCT is 1.46 mPa·s.

Kilic et al. [63] synthesized a series of aromatic acrylate–fluoroacrylate copolymer supercritical CO_2_ thickeners, and studied their structure and mechanism of thickening supercritical CO_2_. Research results show that the thickening property of this copolymer increases firstly and then decreases as the content of aromatic acrylate groups increases. The best solution is a 29% phenyl acrylate–71% fluoroacrylate copolymer. In the supercritical CO_2_ system, the copolymer only needs 5 wt% to increase the viscosity of the system by 205 times at 21.85 °C and 41.4 MPa. At the same time, it was also found that 26% phenyl acrylate (PHA)–74% fluoroacrylate (FA) has a better thickening effect than 27% CHA (cyclohexyl acrylate)–74% FA (fluoroacrylate). This proves that π–π stacking between aromatic rings plays a crucial role in thickening supercritical CO_2_.

In addition, Goicochea et al. [54], also used molecular simulation to study the thickening properties of polymer HFDA. Research shows that the thickening principle of fluorinated polymers mainly has two aspects. On the one hand, the fluorocarbon groups in the molecules can effectively enhance the CO_2_–philic properties of the polymers; on the other hand, the coupling mechanism between polymer molecules, which is the π–π association between styrenes, is stronger than the intramolecular interaction, making it difficult for polymer molecules to diffuse and aggregate, hindering the flow of CO_2_ molecules. This further enhances the thickening property of the supercritical CO_2_ system.

At present, according to the above research results, it can be seen that fluorine–containing polymer thickeners have impressive characteristics in terms of CO_2_–philic properties and CO_2_–thickening properties. However, the economic cost of such fluorinated polymers is too high, and they cannot be metabolized by organisms in the ecosystem. At the same time, they can also cause varying degrees of damage to organisms, such as weakening germ cell activity, interfering with enzyme activity, and damaging cell membrane structures, and so on [22]. Nevertheless, the research on this type of polymer provides theoretical guidance for the future design of economical, environmentally friendly, and pollution–free supercritical CO_2_ thickeners.

### 3.4. Silicone Polymer

Silicone polymers show reliable performance in thickening and are also pollution–free [64], and thus they can be an ideal potential supercritical CO_2_ thickener.

Bae et al. [65,66] used polydimethylsiloxane (PDMS) as a thickener to thicken supercritical CO_2_. Research shows that at 54 °C and 17.2 MPa, the viscosity of the 4% PDMS thickener + 20% toluene cosolvent + 76% liquid CO_2_ system increases to a maximum of 1.2 mPa·s. Compared with pure supercritical CO_2_, the viscosity increased by 30 times. But the disadvantage is that a large amount of cosolvent needs to be added. Zhao et al. [67] also used PDMS to thicken supercritical CO_2_, and the difference was that kerosene was used as a cosolvent because kerosene has a better solubilizing effect than toluene. Research results show that at 51.85 °C, the viscosity of the 5% PDMS thickener + 5% kerosene cosolvent + 90% liquid CO_2_ system increases to 4.67 mPa·s, which makes an increase of 54 times, while the amount of cosolvent is reduced.

Fink et al. [68] studied the feasibility of side–chain functionalization to improve the thickening properties of silicone polymers. The results show that silicone polymers with the appropriate amounts of side–chain functionalization act similarly to fluorinated polyether materials in supercritical CO_2_. Kilic et al. [68,69] enhanced the solubility of PDMS in supercritical CO_2_ through functionalization with propyldimethylamine. O’Brien et al. [70] synthesized a series of aromatic amidated polydimethylsiloxane (PDMS), as shown in Figure 5 of Ref. [70], and studied their solubility and thickening properties in supercritical CO_2_. Research results show that PDMS with anthraquinone–2–carboxamide (AQCA) end groups can thicken supercritical CO_2_ with hexane as a cosolvent, as shown in Figure 6 of Ref. [70]. The reason is that the content of CO_2_–philic groups and benzene ring groups in PDMS containing AQCA is low, and hexane is needed to thicken supercritical CO_2_.

**Figure 2:**
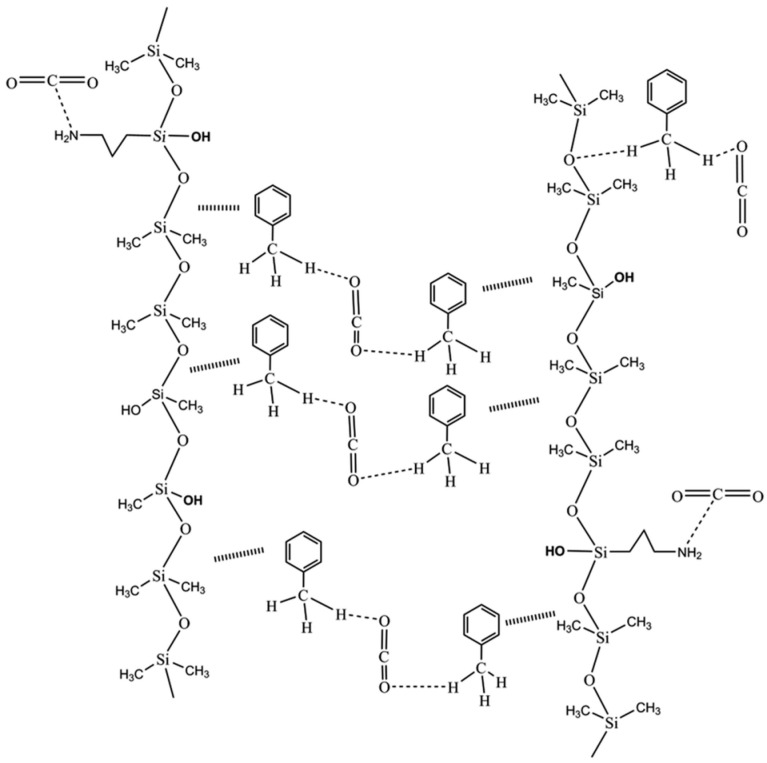
Thickening mechanism of silicone terpolymer [71].

Li et al. [71] synthesized a silicone terpolymer using 0.09 g tetramethylammonium hydroxide catalyst and a molar ratio of aminopropyltriethoxysilane and methyltriethoxysilane of 2:1. At 35 °C and 12 MPa, 3 wt% silicone terpolymer and 7 wt% toluene can thicken the viscosity of the supercritical CO_2_ system by 5.7 times. The mechanisms of silicone terpolymer and toluene are shown in Figure 2. CO_2_ interacts with amino groups. Specifically, N in the amino groups donates electrons to C in the CO_2_ and CO_2_ is located above N. Hydroxyl enhances the stability of the spatial network structure formed by siloxane and CO_2_ molecules. The reason why this type of polymer can thicken supercritical CO_2_ is that the hydroxyl group enhances the spatial network structure. Additionally, the chain structure generated by intermolecular interactions also plays a certain binding role, thereby increasing the flow resistance of CO_2_.

Wang et al. [72] synthesized epoxy–terminated polydimethylsiloxane, as shown in Figure 1 of Ref. [72], and studied its thickening performance in supercritical CO_2_. Research results show that when the shear rate increases, the polymer network structure will be destroyed by shear, and the viscosity of the system also decreases, that is, shear thinning. When the temperature rises, the activity and migration rate of various molecules in the system will be enhanced, which will weaken the intermolecular interaction, thereby destroying the network structure of the polymer and resulting in a decrease in the viscosity of the system. When the pressure increases in the range of 8–14 MPa, the degree of damage to the polymer’s spatial network structure will decrease and the viscosity will increase.

Shen et al. [6] used benzoyl peroxide as the initiator and synthesized a graft copolymer of methylsilsesquioxane and vinyl acetate through graft polymerization, as shown in Figure 3 of Ref. [6]. The thickener does not contain fluorine. Studies have shown that the grafting of PVAc enhances the solubility of siloxane polymers in supercritical CO_2_, and what plays a thickening role would be the network structure generated by polymethylsilsesquioxane. This research provides ideas for solving the solubility problem of polymers in supercritical CO_2_.

## 4. Thickening Mechanism

To obtain an ideal thickener, it should have a certain amount of CO_2_–philic groups (ether groups, carbonyl groups, acetate groups, acetyl groups, sugar ester groups, etc.) and CO_2_–phobic groups in the molecule. CO_2_–philic groups contribute to improve the solubility of the thickener, while CO_2_–phobic groups enhance the viscosity of supercritical CO_2_ through intermolecular association.

The introduced chain–like CO_2_–philic groups should have good flexibility, low cohesive energy, and high free volume. CO_2_–phobic groups can associate or their chains can cross and entangle with each other to form a spatial network structure to restrict the flow of CO_2_ molecules [32]. According to the results of Sagisaka et al. [73], at a certain concentration, the surfactant self–assembles to form linear or rod–like micelles that would intertwine with each other, forming a network structure and increasing the viscosity of CO_2_. It was also observed that rod–like reverse micelles, with different length–to–diameter ratios, exhibit varying thickening effects on supercritical CO_2_ at the same temperature and pressure. For instance, at 45 °C and 350 bar, anisotropic reverse micelles of about 5 to 7 wt% with rod lengths of approximately 166 Å and 583 Å increase the viscosity by 24% and 200%, respectively. Meanwhile, these two groups cannot be too many or too few. If there are too few CO_2_–phobic groups, the solubility of the thickener would be insufficient to achieve the desired thickening effect; while too many CO_2_–phobic groups would also affect the solubility of the thickener [27]. Kilic et al. [63] showed that the thickening properties of aromatic acrylate–fluoroacrylate copolymers exhibited an increase and then a decrease with the content of aromatic acrylate groups. Copolymers containing 29% phenyl acrylate and 71% fluoroacrylate were found to be the most desirable. The addition of only 5 wt% of the copolymer could increase the viscosities of supercritical CO_2_ by up to a factor of 205. Thus, research seeking an optimal ratio or dosage is still needed.

Generally, for surfactant thickeners, one end should be soluble in CO_2_ and the other end should be soluble in water or organic solvents to reduce the surface tension of water or organic solvents in CO_2_. At the same time, to form reverse micelles, two conditions should be satisfied, one is the multiple branched non–polar tail chain and low cohesive energy density, and the other one is a hydrogen–bonding interaction between the polar head group and water [74].

Hydrocarbon thickeners should meet two characteristics. On the one hand, they require large free volume, high chain flexibility, small steric resistance, weak interaction, low glass transition temperature and small steric hindrance, which help the polymer dissolve in CO_2_. On the other hand, polymer chains can cross and entangle with each other to form a spatial network structure, which hinders the flow of CO_2_ molecules and thicken CO_2_ [27].

Fluorine–containing polymer thickeners are obtained by fluorination of hydrocarbon polymers. They are usually weakly polar and have dipole–quadrupole interactions with CO_2_ molecules. At the same time, molecular chains can cross and entangle with each other to form a spatial network structure.

Silicone thickeners generally require cosolvents to improve solubility and thickening effects. The π–π stacking between phenyl groups produces intermolecular interactions, which has the thickening effect of supercritical CO_2_.

For thickening properties of thicker in supercritical CO_2_, in addition to the molecular structure, ratio of CO_2_–philic groups and CO_2_–phobic groups mentioned above, temperature, pressure, thickener molecular weight, and so on are also important influencing factors. The temperature and pressure conditions vary across different reservoir depths, leading to differences in the corresponding properties. The viscosity of supercritical CO_2_ exhibits changes in response to temperature variations. Much research has been conducted [18,75] on the effects of temperature and pressure on CO_2_ viscosity, and the results reveal a decreasing trend in CO_2_ viscosity with increasing temperature, while an there is an increasing trend with increasing pressure.

## 5. Thickening Supercritical CO_2_ in Porous Media

### 5.1. The Flow of CO_2_ in Porous Media

The displacement process of CO_2_ in heterogeneous porous media is one of the most important mechanisms [76]. Fluid physical parameters will cause phase flow instability during the CO_2_ displacement process. Research shows that when CO_2_ is injected into deep salt–water layers, it will displace the pores in a supercritical state. The dominant force in the displacement process is viscous force, and it will affect the form and distribution of fluid flow during the displacement process [77]. The simulation results show that under the condition of low–viscosity enhancement, the displacement process is obviously unstable, and the whole process has a relatively obvious fingering phenomenon; on the contrary, under the condition of higher viscosity enhancement, the displacement process is more stable, and no obvious fingering phenomenon occur [78].

### 5.2. Adsorption in Porous Media

During CO_2_ fracturing, CO_2_ thickeners may remain in the shale reservoir, and these chemicals may pollute the reservoir environment. Therefore, there is the need for the low adsorption of CO_2_ thickeners in porous media in practical industrial applications. If the adsorption within the porous medium is excessive, it may lead to the blockage of the pores [58]. Afra et al. [23] conducted experiments on a variety of thickeners currently available on the market, and the results showed that several thickeners containing the element fluorine showed significant adsorption to the surface of porous media [23]. They also used MD simulations to study the adsorption problem and came up with the agreement between the simulation and experimental results. They proposed an effective theoretical approach to study the adsorption of thickeners in porous media [23]. Li et al. [79] modified the thickener and prepared a new type of PDMS, and then investigated its contact angle. The results showed that the contact angle of PDMS decreased from 138° to 99° with increasing temperature, with a significant decreasing trend, while the contact angle of the prepared novel PDMS decreased from 135° to 127° [79]. Compared with the two, the novel PDMS has less adsorption on the reservoir surface, which is more favorable to reduce the contamination of the thickener on the reservoir. 

In conclusion, the thickener should not exhibit excessive adsorption on the surface of porous media, as it would lead to a poorer process, economic problems, and excessively large reductions in permeability due to wettability alteration. If the thickener is brine–soluble (which is unlikely, given the low mutual solubility of CO_2_ and water), the thickener may separate out into the brine within the porous media. If the thickener is crude–oil–soluble, a portion of the thickener may ultimately contaminate the crude oil product and potentially cause problems in downstream processing equipment within refineries [23].

## 6. Summary and Outlook

This article provides a comprehensive review of four types of polymer thickeners, namely surfactants, hydrocarbons, fluorine–containing polymers, and silicones. We focused on analyzing their solubility and thickening characteristics in supercritical CO_2_ systems, and also explained the thickening mechanisms. Furthermore, we discussed the flow and adsorption of thickeners in porous media.

For surfactants, the thickening property is adequate, while solubility is far from satisfactory [39,43]. For example, a 1 wt% surfactant tributyltin fluoride thickener and a 40–45 wt% pentane cosolvent can thicken the viscosity by 10–100 times [39]. However, the solubility of the thickener is poor, requiring a significant amount of cosolvent or CO_2_–philic groups. In addition, silicones show similar solubility and thickening characteristics to surfactants, where 5% PDMS thickener with a small amount of cosolvent increases the viscosity to 4.67 mPa·s, which is an increase of up to 54 times [67]. The economic cost and environmental problems of cosolvents have become an urgent issue to be addressed.

Among the four thickeners, fluorinated thickeners have the most outstanding thickening properties, brought about by the interactions between the fluorine element and CO_2_. According to our research, the addition of 5 wt% PolyFast could increase the viscosity of supercritical CO_2_ by up to 400 times [67], which is the highest on record so far, according to our knowledge. Meanwhile, this copolymer also has fantastic solubility under reservoir conditions. However, this type of thickener is not commonly used, mainly because of its economic cost and biological toxicity. At present, most other polymer thickeners still require cosolvents to thicken liquid CO_2_; however, it is not environment friendly.

It was found that PVAc is one of the optimal CO_2_–philic hydrocarbon homopolymers because of the acetic acid group [73], yet its thickening properties are not ideal at present. However, PVAc is an ideal economical and environmentally friendly thickener, and an abundance of research has been conducted to improve the viscosity with it [32,52,53], such as forming a binary copolymer or spatial network structure, etc. This has made the PVAc–based system a mainstream thickener in work sites.

In recent years, the thickening mechanism and promotion of thickeners have been investigated through molecular modeling of polymer–CO_2_ systems. Regarding the thickening mechanism, it has been recognized that CO_2_–soluble polymers may have a moderately branched structure, high free volume, low solubility parameter, and contain Lewis acid–base groups. By introducing CO_2_–philic groups, the interaction between the thickener molecules and CO_2_ can be facilitated, thereby increasing the solubility of the thickener in supercritical CO_2_. The polymers should also contain CO_2_–phobic groups, which can combine with neighboring CO_2_–phobic groups to form a viscosity–enhancing network structure. Furthermore, the thickeners should exhibit low adsorption onto rock to minimize the blockage of rock pores, maintain the fluidity of the fracturing fluid, and reducing pollution and damage to the rock environment. Therefore, further research may focus on these aspects, addressing economic and technological barriers, as well as environmental concerns. The development of efficient, environmentally friendly, and cost–controllable thickeners can help promote engineering site applications.
